# Effects of Different Zn^2+^ Concentrations and High Hydrostatic Pressures (HHP) on Chlorophyll Stability

**DOI:** 10.3390/foods11142129

**Published:** 2022-07-18

**Authors:** Yuwei Hu, Hongnan Sun, Taihua Mu

**Affiliations:** Laboratory of Food Chemistry and Nutrition Science, Institute of Food Science and Technology, Chinese Academy of Agricultural Sciences, Key Laboratory of Agro-Products Processing, Ministry of Agriculture and Rural Affairs, No. 2 Yuan Ming Yuan West Road, Haidian District, P.O. Box 5109, Beijing 100193, China; huyuwei0520@126.com (Y.H.); mutaihua@126.com (T.M.)

**Keywords:** Zn^2+^ replacement rate, chlorophyll structure, color, antioxidant activity, high hydrostatic pressure online fluorescence, thermal stability

## Abstract

This study provides a new idea for improving chlorophyll stability and color quality of green leafy vegetables by Zn^2+^ synergistic HHP. Zn-chlorophyll was prepared with zinc acetate and chlorophyll under HHP treatment. The effects of different zinc acetate concentrations and pressures on chlorophyll color, antioxidant activity, Zn^2+^ replacement rate, structure, and thermal stability were analyzed. Results showed with increased zinc acetate concentration and pressure, −a* value, antioxidant activity, and Zn^2+^ replacement rate of samples gradually increased. However, FTIR indicated the structure did not change. HHP fluorescence online analysis showed fluorescence intensity of samples decreased with zinc acetate concentration and pressure increasing. With zinc acetate 10 mg/100 mL and HHP 500 MPa, the highest −a* value (5.19), antioxidant activity (ABTS, DPPH, and FRAP were 37.03 g ACE/100 g, 25.95 g ACE/100 g, 65.43 g TE/100 g DW, respectively), and Zn^2+^ replacement rate (42.34%) were obtained. Thermal stability of Zn-chlorophyll obtained by synergistic effect was improved significantly.

## 1. Introduction

In recent years, natural pigments have become increasingly popular in the food industry due to their health benefits, green color, and consumption safety. Among them, chlorophyll is a tetrapyrrole pigment found in common green fruits and vegetables, and contains a variety of physiologically active functions, such as anti-oxidation, hematopoiesis, maintaining enzyme activity, detoxification, disease resistance, vitamin source and so on [1,2]. However, natural chlorophyll is less stable, and light, oxygen, high temperature, and enzymes can degrade it and produce some unpleasant colors compared with synthetic green color additives [3]. The color of food is one of the important sensory quality indicators. People often first judge the merits of food by color before receiving other information, to decide on the “choice” of a particular food [4]. Due to the thermal instability of chlorophyll, many food processing methods such as blanching and drying, etc., will cause a chlorophyll demagnetization reaction, and the product to turn from green to brown, which seriously affects the sensory quality of the commodity [5]. At present, the main methods often used to improve the stability of chlorophyll are (i) Inhibition of enzyme activity. Using high-temperature blanching, and other means to inhibit the activity of enzymes related to the degradation of chlorophyll [6]. (ii) Alkalization of green protection. Using alkaline substances to increase the pH of the product, chlorophyll is more stable in an alkaline environment [7,8]. (iii) Ion replacement. Chlorophyll derivatives of copper and zinc complexes are significantly more stable than chlorophyll, and can maintain the color of green vegetables [9,10]. However, the high-temperature blanching process leads to the loss of a large number of nutrients, and the high temperature accelerates the demagnetization of chlorophyll [11]. With the extension of storage time, the protective effect of alkalinization will gradually fail. In addition, the copper ion is a type of heavy metal, and the human body cannot consume too much. Nevertheless, using zinc ions for ion replacement is a good choice. The tolerable upper limit of intake is 40 mg/day in adults [12]. The introduction of zinc ions not only improves chlorophyll’s tolerance to acid, heat, etc., but also has a beneficial effect on human health with moderate intake, which can strengthen the immune system [13].

High hydrostatic pressure (HHP) is a green, safe, stable, and efficient non-thermal food processing technology developed in recent years. It can provide instant and uniform pressure at every point of the material. Once the set pressure is reached, no more energy input is needed to maintain the high-pressure state, to achieve material modification and food quality improvement in a relatively short period [14,15]. HHP has been used to improve the stability of chlorophyll in food. However, most of the studies have attributed the improvement of chlorophyll stability by HHP to the inhibitory effect of pressure on the enzymatic activities of chlorophyllase and polyphenol oxidase [16,17]. Although these studies are based on the food system, due to the complexity, it is impossible to explain the direct effect of HHP on chlorophyll. Furthermore, the effect of Zn^2+^ replacement and HHP synergy on chlorophyll stability and its mechanism of action has not been reported, and we used an HHP online monitor coordinated with a fluorescence spectrophotometer to observe the changes of chlorophyll during pressurization.

In the present study, Zn-chlorophyll was prepared at room temperature, pH = 5.5, different concentrations of zinc acetate (0, 4, 7, and 10 mg/100 mL) and different HHP (0.1, 100, 300, and 500 MPa). The electronic eye, fluorescence spectrophotometer, high-performance liquid chromatography (HPLC), and Fourier transform infrared (FTIR) were used to investigate the effects of different Zn^2+^ concentrations and pressures on the color, antioxidant activity, Zn^2+^ replacement rate, and structure of chlorophyll samples. HHP online monitor coordinated with fluorescence spectrophotometer was used to monitor the fluorescence patterns of chlorophyll samples during pressurization at different Zn^2+^ concentrations and pressures. In addition, the thermal stability of chlorophyll samples co-treated with Zn^2+^ and HHP in an acidic environment (pH = 5.5) was also investigated. It is hoped that the stability of chlorophyll can be improved by HHP and Zn^2+^. Moreover, we hope to develop an efficient and environmentally friendly method to improve the stability of chlorophyll and explain its mechanism.

## 2. Materials and Methods

### 2.1. Materials

Chlorophyll *a* (≥85%) was purchased from Macklin Bio-Technology Co., Ltd., (Shanghai, China). Chlorophyll *b* (≥90%) was purchased from OKA Bio-Technology Co., Ltd., (Beijing, China). All chemicals, solvents, and reagents used in the experiments were at least of analytical grade.

### 2.2. Sample Preparation

Chlorophyll *a* and chlorophyll *b* were dissolved into 75% ethanol in a 2:1 ratio, and the chlorophyll concentration was controlled at 9 mg/100 mL. Aqueous zinc acetate solutions with concentrations of 0, 4, 7, and 10 mg/100 mL were prepared, and mixed together with the chlorophyll solution, respectively. The pH values of the mixtures were adjusted to 5.5 with HCl or NaOH.

Consequently, the above prepared mixtures were pressurized in a hydrostatic pressurization unit (model HHP.L2-600/10, Tianjin Huataisenmiao Engineering and Technique Co., Ltd., Tianjin, China), with a maximum pressure of 600 MPa. Distilled water was used as the pressure media. The temperature in the processing vessel was approximately 25 °C. The samples were treated at 0.1, 100, 300, and 500 MPa for 10 min, respectively. Then the color, antioxidant activity, Zn^2+^ replacement rate, and structure characteristics of the chlorophyll samples were analyzed immediately.

### 2.3. 100 °C Thermal Treatment

For thermal stability analysis, the chlorophyll samples treated with Zn^2+^ in conjunction with HHP in the previous step were heated at 100 °C for 10 min in a water bath (Beijing Tianlin Hengtai Technology Co., Ltd., Beijing, China), followed by rapid cooling in an ice bath. The color, antioxidant activity, and structure characterization of the thermal-treated chlorophyll samples were analyzed immediately.

### 2.4. Color Measurement

Color analysis was carried out using an electronic eye (Verivide, UK). In the electronic eye analysis, the color calibration of the electronic eye equipment was firstly performed using the calibration white plate and standard color plate, and then 3 mL of sample solutions with different concentrations of Zn^2+^ solubility and HHP treatment were poured into disposable Petri dishes and placed into the order of advance calibration. The high-resolution digital photographs were taken with an electronic eye on the observation plate. The color differences of the samples were analyzed using red and green values a* (+a* and −a* are red and green, respectively).

### 2.5. Antioxidant Activity Determination

#### 2.5.1. ABTS+• Radical Scavenging Assay

The ABTS+• radical scavenging capability assay (ABTS) was adopted based on the method of Bae et al. [18] with little modification. The ABTS+• solution was prepared with 7 mM ABTS diammonium salt solution and 2.45 mM K_2_S_2_O_8_ solution overnight at room temperature in the dark for 12 h. The mixture was diluted approximately 50-fold with ethanol, and the absorbance at 734 nm was controlled to 0.70 ± 0.02. The 1 mL sample solution (the blank control used ethanol instead of the sample) was added to 2 mL ABTS+• solution followed by incubation at room temperature for 6 min in the dark, and the absorbance was read at once at 734 nm. The results were expressed as ascorbic acid equivalent (ACE) relative to sample weight (g ACE/100 g DW).

#### 2.5.2. DPPH Radical-Scavenging Assay

The procedure used for the DPPH radical (DPPH) scavenging activity assay was that described by Makori et al. [19], with slight modifications. Briefly, 0.2 mM of DPPH solution (2 mL) was prepared and reacted with 2 mL of sample solution. The reaction was kept in a dark room for 30 min at room temperature. Absorbance readings were recorded using a spectrophotometer at 517 nm. The results were expressed as ascorbic acid equivalents (ACE) on a DW basis (g ACE/100 g DW).

#### 2.5.3. Ferric Reducing Antioxidant Power (FRAP) Assay

The FRAP was adopted based on the method of Makori et al. [20] with little modification. To obtain a FRAP solution, 10 mmol/L TPTZ solution (dissolved by 40 mmol/L HCl solution), 20 mmol/L FeCl_3_ solutions (dissolved by acetate buffer), and 0.3 mol/L (pH = 3.6) acetate buffer solution were mixed in a volume ratio 1:1:10, and the mixed solution was incubated at 37 °C for 30 min. The 0.15 mL sample solution (the blank control used distilled water instead of the sample) was added to 2.85 mL FRAP solution followed by incubation at room temperature for 30 min in the dark, after that the absorbance was measured immediately at 593 nm. The results were expressed as Trolox equivalent (TE) relative to sample weight (g TE/100 g DW).

### 2.6. Zn^2+^ Replacement Rate Analysis

Quantification of the replacement rate of Zn^2+^ (%) was achieved by HPLC (RP-HPLC, Shimadzu LC20A, Kyoto, Japan) according to the method of Das et al. [21] with little modification. Separations were conducted at 28 °C on column C18 (150 mm × 4.6 mm; 5 µm particle size). Briefly, the mobile phase (A) consisted of 1 M ammonium acetate and methanol with ratio 20:80 (*v*/*v*); and the mobile phase (B) was acetone and methanol with ratio 20:80 (*v*/*v*); the HPLC gradient program was 0 min, 0% B; 15 min, 100% B; 25 min, 100% B; 28 min, 0% B and then 32 min 0% B. The flow rate was 1 mL per minute and the injection volume was 50 μL. The sample was filtered through a 0.22 um membrane. Quantitative analysis was performed using the regression equations obtained for each standard compound. Pheophytin a and pheophytin b were used by adjusting the pH of chlorophyll a and chlorophyll b to 3.0 and leaving them for 12 h. The chromatograms of chlorins were recorded at 665 nm.

### 2.7. Structure Characterization Analysis

#### 2.7.1. FTIR Analysis

FTIR spectra of the samples were recorded using FTIR spectrometer (Tensor-27; Bruker, Karlsruhe, Germany) according to Mathiyalagan et al. [22] with little modification. Briefly, clean the place where the sample is placed with alcohol cotton. After the alcohol is completely volatilized, drop about 10 uL of the sample solution in the middle and analyze it in an FTIR machine. FTIR spectra ranging from 4000 to 400 cm^−1^ were recorded with a resolution of 4 cm^−1^ and the FTIR data were plotted using Origin 8.5 software.

#### 2.7.2. HHP Fluorescence Online Analysis

Equal volumes of chlorophyll solution were mixed with different concentrations of zinc acetate solution and the pH was adjusted to 5.5. The analysis was performed with an HHP online monitor (model TNV-1100-7000, Shenzhen Telide Fluid System Co., Ltd., Guangdong, China) with a maximum pressure of 500 MPa cooperated with fluorescence spectrophotometer (F-4600, Hitachi, Japan). The samples were treated at 0.1, 100, 300, and 500 MPa for 10 min, respectively. Next, fluorescence analysis was performed at 0, 2, 4, 6, 8, and 10 min of the HHP treatment. The excitation (EX) slit width and emission (EM) slit width are 5 nm and 20 nm, respectively, in the EX wavelength of 475 nm and EM wavelength of 350~750 nm. Moreover, the EX slit width and EM slit width were both 20 nm in the range of EX wavelength 350–550 nm and EM wavelength 300–750 nm with a scanning speed of 1200 nm/min tracing speed force and voltage 400 V. The 3-dimensional (3D) fluorescence contour spectra were recorded after HHP treatment.

#### 2.7.3. Fluorescence Scanning

Fluorescence scanning of chlorophyll samples was performed after thermal stabilization treatment. The fluorescence spectrum was measured on the fluorescence spectrophotometer (F-2500, Hitachi, Japan) according to [23] with little modification. The EX wavelength is 475 nm, the EM wavelength is 350–750 nm, the scanning speed is 300 nm/min, and the EX and EM slit width is 5 nm. All experiments were carried out at room temperature.

### 2.8. Statistical Analysis

All the experiments were conducted in triplicate, and the results were expressed as means ± standard deviation. Statistical analysis was executed using the Statistic Package for Social Science 21.0 (SPSS 21.0), ANOVA tests, followed by Duncan‘s multiple range test, and *p* < 0.05 was considered statistically significant.

## 3. Results and Discussion

### 3.1. Color Analysis

**Figure 1:**
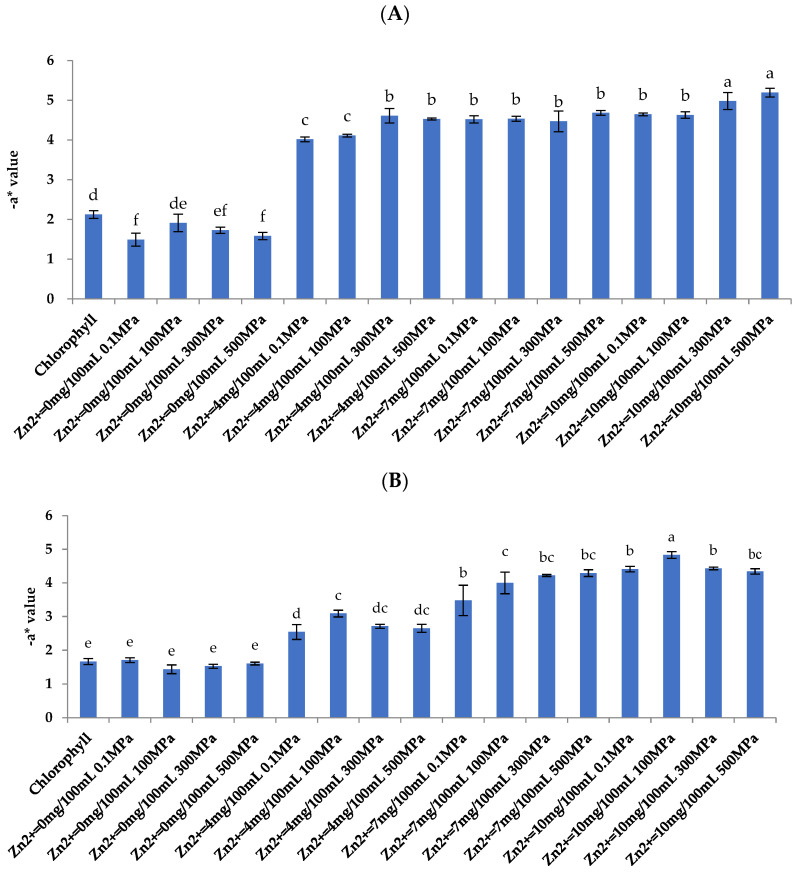
Electronic eye color analysis of samples after different concentrations of Zn^2+^ and high hydrostatic pressure (HHP) treatment (**A**), and after different concentrations of Zn^2+^, HHP, and 100 °C thermal treatment (**B**). Different letters on top of the bars indicate significant difference at *p* < 0.05. Error bars show standard deviations of the mean (N = 3).

It can be seen from Figure 1A and Supplementary Figure S1 that when pH = 5.5 and Zn^2+^ concentration is 0 mg/100 mL, the −a* value of chlorophyll samples (0.1, 100, 300, and 500 MPa) is significantly lower than that of chlorophyll solutions without any treatment (2.12). This is mainly because the structure of chlorophyll is unstable in an acidic environment, and chlorophyll (green) is degraded and produces magnesium chlorophyll (brown) [24]. With the introduction of Zn^2+^, the –a* value of the chlorophyll sample increased (4.02–5.19), which is significantly higher than that of chlorophyll solution without any treatment (2.12). Under the same Zn^2+^ concentration, the green degree of the sample treated with high pressure (100, 300, and 500 MPa) is better than that of the chlorophyll sample under 0.1 MPa. When the Zn^2+^ concentration is 10 mg/100 mL and the pressure is 500 MPa, the green degree of the sample is the deepest (5.19), which increased by about 144.81% compared with the control group (2.12). 

The results showed that the color of the chlorophyll samples was greatly improved after being treated with Zn^2+^ and HHP. Meanwhile, the increase in −a* value compared to the untreated chlorophyll solution demonstrated the production of Zn-chlorophyll. Senklang and Anprung [25] found that Zn-chlorophyll is more stable than chlorophyll. Previous studies reported that Zn-chlorophyll complexes have a shorter bond length between metal ions and tetrapyrrole rings than natural chlorophyll, resulting in higher bond energy and higher stability [26,27]. Our results also confirmed this conclusion, Zn-chlorophyll samples remained green in an acidic environment (pH = 5.5), and its stability is better than that of chlorophyll.

### 3.2. Antioxidant Activity

**Figure 2:**
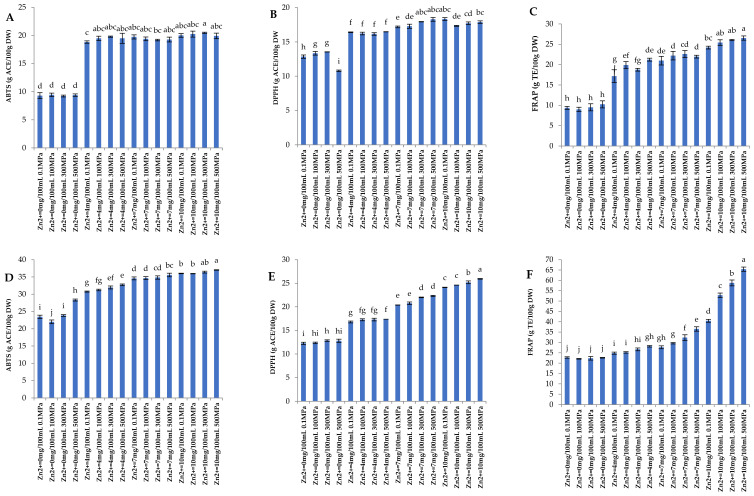

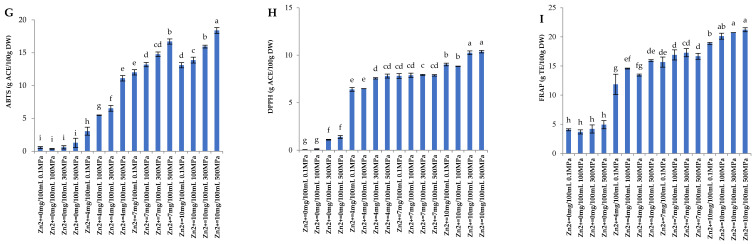
Antioxidant activity of samples before different concentrations of Zn^2+^ and high hydrostatic pressure (HHP) treatment (**A**–**C**), after different concentrations of Zn^2+^ and HHP treatment (**D**–**F**), and after different concentrations of Zn^2+^, HHP, and 100 °C thermal treatment (**G**–**I**). Different letters on top of the bars indicate significant difference at *p* < 0.05. Error bars show standard deviations of the mean (N = 3).

Chlorophyll has a variety of physiological activities, among which antioxidant activity is a very important characteristic [28]. From Figure 2, under the same concentration of Zn^2+^, the antioxidant activity of chlorophyll samples treated with Zn^2+^ synergistic HHP is the strongest, followed by that before Zn^2+^ synergistic HHP treatment, and finally after Zn^2+^ synergistic HHP treatment and 100 °C −10 min thermal treatment. ABTS, DPPH, and FRAP show the same trend. Before Zn^2+^ synergistic HHP treatment, ABTS free radical scavenging activity, DPPH free radical scavenging activity, and FRAP were 9.24–20.47 g ACE/100 g DW, 10.79–18.34 g ACE/100 g DW and 9.01–26.54 g TE/100 g DW, respectively. Although the HHP treatment was not carried out, the antioxidant activity of the treatment group with high Zn^2+^ concentration is higher than that of the treatment group with low Zn^2+^ concentration. After Zn^2+^ synergistic HHP treatment, ABTS free radical scavenging activity, DPPH free radical scavenging activity, and FRAP were 22.01–37.03 g ACE/100 g DW, 12.27–25.95 g ACE/100 g DW and 22.10–65.43 g TE/100 g DW, respectively. Compared with that before HHP treatment, the antioxidant activity of the sample has been significantly improved. After Zn^2+^ synergistic HHP treatment and 100 °C −10 min thermal treatment, ABTS free radical scavenging activity, DPPH free radical scavenging activity, and FRAP were 0.37–18.40 g ACE/100 g DW, 0.05–10.37 g ACE/100 g DW and 3.70–21.23 g TE/100 g DW, respectively. Compared with before the synergistic treatment, the antioxidant activity of chlorophyll samples after Zn^2+^ and HHP treatment was significantly increased, indicating the formation of Zn-chlorophyll during the process, and there was a positive correlation with the concentration of Zn^2+^ and pressure.

From the results, it can be concluded that the antioxidant activity of chlorophyll samples treated with Zn^2+^ and HHP in an acidic environment (pH = 5.5) was significantly increased. In addition, there was a significant positive correlation between Zn^2+^ concentration and pressure. With the increase of Zn^2+^ (0, 4, 7, and 10 mg/100 mL), ABTS free radical scavenging activity, DPPH free radical scavenging activity, and FRAP increased by about 115.78%, 41.04% and 167.94%, respectively. When the Zn^2+^ concentration was 10 mg/100 mL, ABTS free radical scavenging activity, DPPH free radical scavenging activity, and FRAP of the chlorophyll samples at 500 MPa increased by about 5.79%, 13.48% and 64.29%, respectively, compared to the 0.1 MPa treated samples.

Suryani et al. [29] studied the antioxidant activity of chlorophyll extracted from pandan (Pandanus amaryllifolius Roxb) leaves, and they found the radical scavenging activity of DPPH and FRAP assay showed that chlorophyll and chlorophyllide extracts exhibited higher activity, followed by pheophytin and pheophorbide. Kang et al. [30] synthesized chlorophyll derivatives, pheophytins, and Zn-pheophytins, from chlorophylls extracted from spinach, characterized them, and evaluated their antioxidant activities, and their findings indicated that Zn-pheophytins have strong antioxidant properties. However, in food-based studies, because the antioxidant activity is not entirely dependent on chlorophyll content and changes in its structure, it is also associated with many other types of compounds, such as polyphenols, flavonoids, and *β*-carotene [31]. 

### 3.3. Zn^2+^ Replacement Rate Analysis

The standard equations and correlation coefficient (R^2^) of chlorophyll *a*, chlorophyll *b*, pheophytin *a* and pheophytin *b* are as follows: y = 54842 × −112111 (R^2^ = 0.9811); y = 30703 × −57135 (R^2^ = 0.9696); y = 12695 × −2289.6 (R^2^ = 0.9953); y = 7780.6 × −2383.5 (R^2^ = 0.9527). HPLC chromatograms of the sample at 665 nm are shown in supplement Figure 2. As can be seen from Table 1, after different Zn^2+^ concentrations and HHP treatment, it can be seen that the retention rate of Zn^2+^ in chlorophyll structure increases significantly with the increase of Zn^2+^ concentration and pressure. Especially when the concentration of Zn^2+^ is 10 mg/100 mL and the pressure is 500 MPa, the replacement rate of Zn^2+^ can reach 42.34%. Under the same Zn^2+^ concentration (4, 7, 10 mg/100 mL), we can find that the replacement rate of Zn^2+^ increases with the addition of pressure. Moreover, when under the same pressure, the Zn^2+^ replacement rate also increased with the increase of concentration. This concludes that in an acidic environment (pH = 5.5), a certain concentration of Zn^2+^ and a certain intensity of HHP have a synergistic effect on the replacement rate of Zn^2+^ in chlorophyll solution. The increase in the Zn^2+^ replacement ratio echoes the increase in greenness in the color of chlorophyll samples. This also confirms that Zn^2+^ replacement with HHP may be a green and environmentally friendly non-thermal processing method to improve chlorophyll stability.

### 3.4. Structure Characterization Analysis

#### 3.4.1. FTIR Analysis

**Figure 3:**
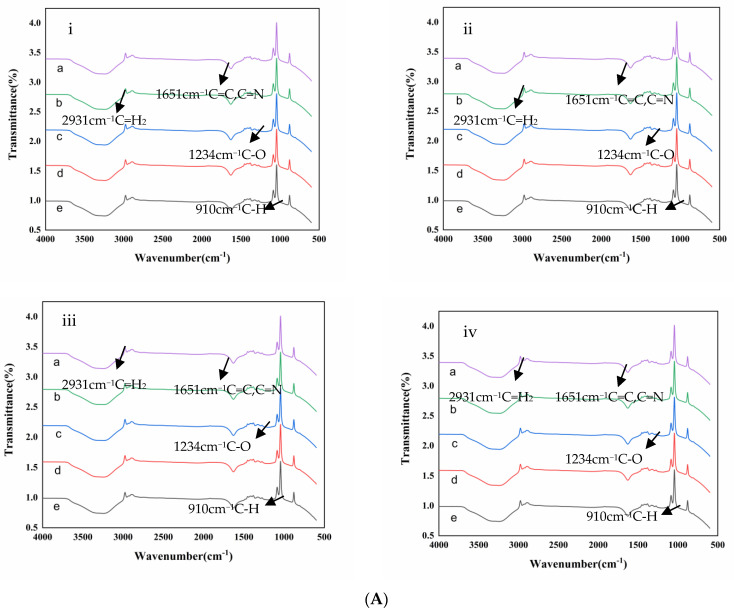

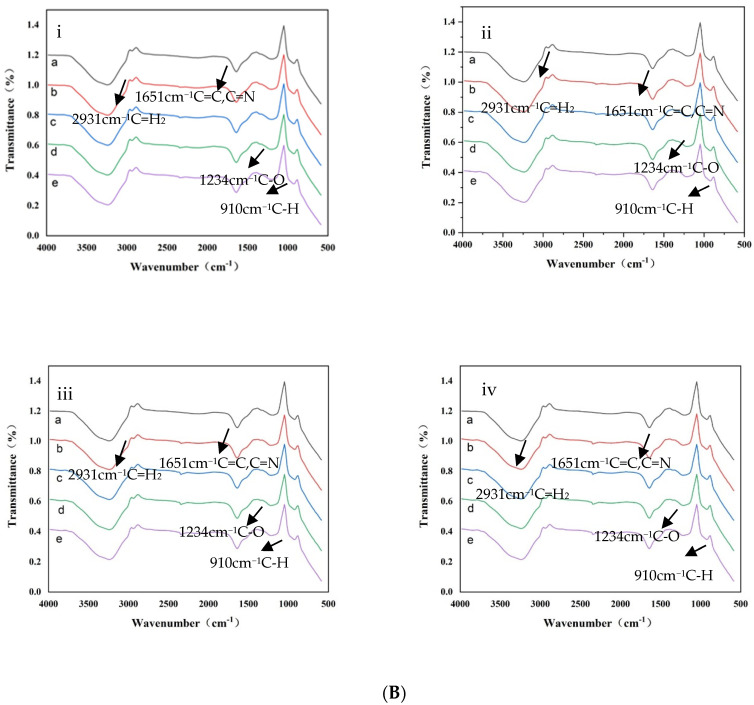
Fourier transform infrared (FTIR) spectroscopy of samples after different concentrations of Zn^2+^ and high hydrostatic pressure (HHP) treatment (**A**), and after different concentrations of Zn^2+^, HHP, and 100◦C thermal treatment (**B**). (**i**–**iv**) represent Zn^2+^ concentrations of 0, 4, 7 and 10 mg/100 mL, respectively, and (a) is chlorophyll, (b), (c), (d) and (e) are 0.1, 100, 300 and 500 MPa treated samples, respectively.

From the FTIR spectrum (Figure 3A,B), it can be seen that the spectra of chlorophyll and Zn-chlorophyll are very similar, there is no obvious difference, regardless of whether 100 °C −10 min thermal treatment is carried out or not. The characteristic bands of chlorophylls appeared at 2931 (C-H stretching in phytol), 1651 (skeletal C=C and C=N stretching of aromatic system in chlorophyll), 1234 (C-O stretching), and 910 (C-H stretching). [22,30]. Our results are similar to those of Mathiyalagan et al. [22], who extracted and purified chlorophyll from Ficus leaves and synthesized metal-chlorophyll complexes. However, some previous studies have shown that there are differences between the FTIR spectra of chlorophyll and metal–chlorophyll. Konwar and Baruah [32] found that the complexation of metal ions with chlorophyll can be observed by the change in the FTIR spectrum at 1540 cm^−1^ for C=C and C=N. Petrović et al. [33] found that zinc–chlorophylls’ C=C, C=N, C-O, and C=O were different from those of magnesium–chlorophylls which were extracted from spinach leaves (*Spinacia oleracea*). This may have occurred since this phenomenon may be based on different substrates. HHP does not change the internal structure of small molecules such as chlorophyll [34]. 

#### 3.4.2. HHP Fluorescence Online Analysis

**Figure 4:**
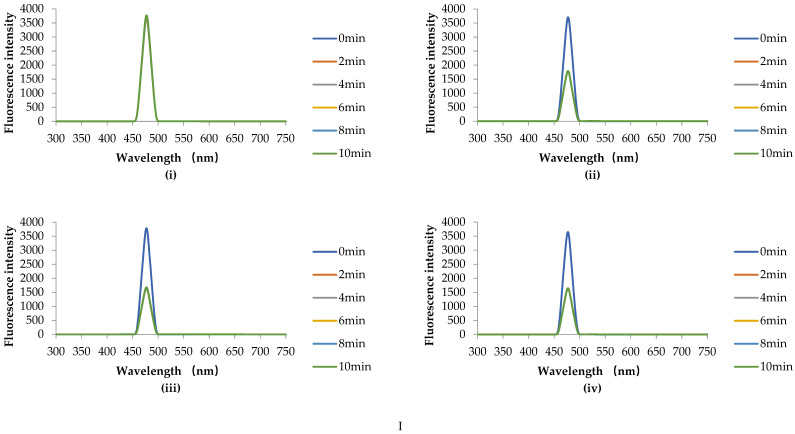

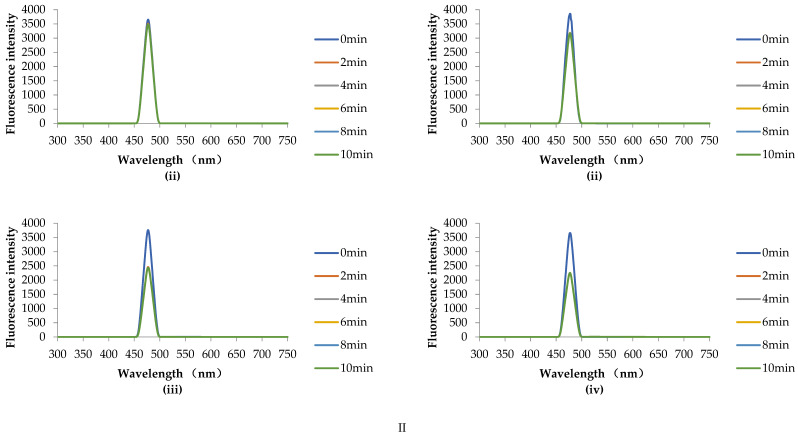

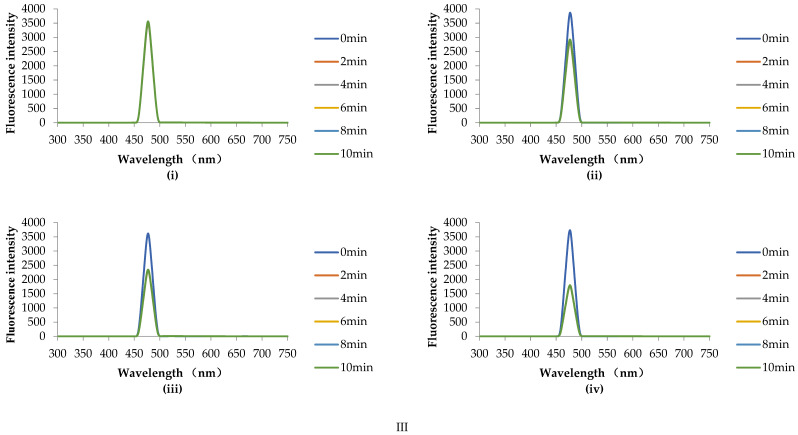

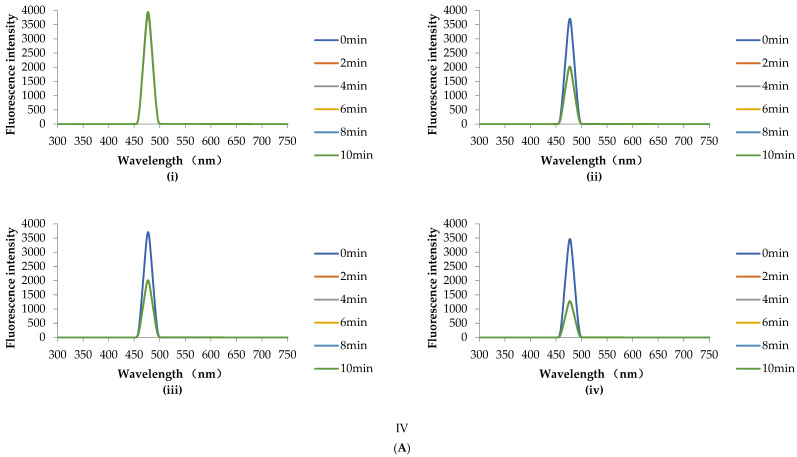

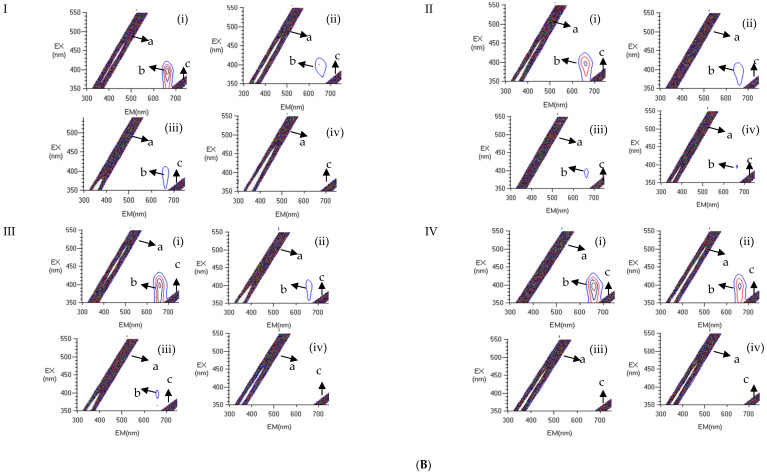
Variation of fluorescence intensity with time (**A**) and 3-dimensional (3D) fluorescence contour spectra of samples (**B**) treated with different Zn^2+^ concentrations and high hydrostatic pressure (HHP) at room temperature, pH = 5.5. **I**–**IV** represent Zn^2+^ concentrations of 0, 4, 7, and 10 mg/100 mL respectively, and (**i**–**iv**) are 0.1, 100, 300 and 500 MPa treated samples, respectively, and a represents the characteristic peak of the Raman spectrum, b represents the fluorophore of chlorophyll, and c represents the Rayleigh scattering peak.

According to Figure 4A, the initial fluorescence intensity of the sample is almost the same at 0 min, the introduction of Zn^2+^ has little effect on the fluorescence intensity of chlorophyll in the absence of HHP treatment. After HHP treatment, the fluorescence intensity of chlorophyll samples decreased significantly, regardless of the presence of Zn^2+^ in the solution. However, the fluorescence intensity of the chlorophyll sample did not change with the extension of HHP treatment time. From Figure 4(AI), it can be clearly seen that when the Zn^2+^ concentration was 0 mg/100 mL and the pressure increased from 0.1 MPa to 100 MPa, the fluorescence intensity of the sample decreased significantly (compared with the 0.1 MPa treatment group). However, the decrease of fluorescence intensity is not particularly obvious with the continuous increase of pressure. From Figure 4(AII–AIV), it can be found that the fluorescence intensity of the sample decreased gradually with the increase of pressure when the Zn^2+^ concentration was 4, 7, and 10 mg/100 mL. When under the same high pressure, the fluorescence intensity of the sample also showed a gradual downward trend with the increase of Zn^2+^ concentration. The same results are also obtained in the 3D fluorescence contour spectra (Figure 4B), where a represents the characteristic peak of the Raman spectrum, b represents the fluorophore of chlorophyll, and c represents the Rayleigh scattering peak. It can be seen from Figure 4B that the fluorescence intensity of chlorophyll decreases with the increase of pressure. When the pressure reaches 500 MPa, the characteristic peak of chlorophyll fluorophore disappears. Previous studies have shown that HHP can denature or inactivate organic macromolecules such as protein and starch, but has no significant effect on the internal covalent bonds of small molecular compounds such as vitamins, pigments, and flavor substances [34,35]. The decrease in fluorescence intensity is a result of the quenching induced by chlorophyll aggregation [36]. Qu et al. [37] showed that chlorophyll *a* formed J-type and H-type aggregation in acidic ethanol solution. Our results also show this conclusion, and HHP will be conducive to the aggregation of chlorophyll.

The conclusion can be drawn that the synergistic effect of HHP and Zn^2+^ can increase the fluorescence quenching of chlorophyll. Based on Figure 4A,B, it can be inferred that the reasons for this phenomenon are as follows: (i) the fluorescence quenching of chlorophyll increased by the introduction of zinc ions in the solution system; (ii) in an acidic environment, due to the intolerance of chlorophyll to acid, magnesium ions are replaced by hydrogen ions, and chlorophyll becomes pheophytin. In the process of HHP treatment, pressure can promote Zn^2+^ to enter the central structure of chlorophyll and form Zn-chlorophyll.

### 3.5. Thermal Stability Analysis

#### 3.5.1. Color Analysis

From Figure 1B, after Zn^2+^ synergistic HHP and 100 °C −10 min thermal treatment, it can be found that the −a* value of all samples decreased in varying degrees compared to the pre-heat treatment (Figure 1A). However, the color of chlorophyll samples treated with Zn^2+^ is still significantly better than that without Zn^2+^ treatment. In addition, the color of the HHP treatment groups was better than that of the 0.1 MPa group. Similarly, with the increase of Zn^2+^ concentration, the −a* value of the sample shows an obvious increasing trend. The −a* value of the HHP treatment group was significantly higher than that of samples with 0.1 MPa treatment, but after thermal treatment, the −a* value of samples did not significantly increase with the increase of pressure. When the Zn^2+^ concentration was 4 and 7 mg/100 mL, the −a* value of 100, 300, or 500 MPa treatment group did not significantly change, while when the Zn^2+^ concentration was 10 mg/100 mL, the −a* value of 100 MPa treatment group was the largest (4.83). Meanwhile, it can be observed that the synergistic effect with HHP when the Zn^2+^ (4, 7, and 10 mg/100 mL) greatly increases the -a* value of the treated samples, compared with the control thermal-treated chlorophyll solution (1.67). Heating usually leads to the green fading of chlorophyll. On the other hand, acid can also accelerate this reaction [38]. However, the co-processed chlorophyll samples remain a bright and attractive green. Thus, the co-treatment greatly improves the thermal stability of the chlorophyll samples in an acidic environment (pH = 5.5). The reason is that Zn-chlorophyll is more resistant to acid and heat than chlorophyll [25]. Nonetheless, long-term (10 min) thermal treatment at 100 °C still caused a slight decrease in the −a* value. 

#### 3.5.2. Antioxidant Activity

According to Figure 2G–I, when Zn^2+^ is 10 mg/100 mL, HHP is 500 MPa, although the heat treatment at 100 °C −10 min reduced its antioxidant activity to a large extent, ABTS free radical scavenging activity, DPPH free radical scavenging activity, and FRAP were 37.03–18.40 g ACE/100 g DW, 25.95–10.37 g ACE/100 g DW and 65.43–21.23 g TE/100 g DW, respectively. The effect of the HHP treatment groups (100, 300, and 500 MPa) was better than that of the 0.1 MPa group, and the effect of adding Zn^2+^ (4, 7, and 10 mg/100 mL) is better than that of Zn^2+^ is 0 mg/100 mL. Meanwhile, this also shows that the ability of the chlorophyll samples to resist acid and heat has been significantly improved after the synergistic treatment of Zn^2+^ and HHP. As previously reported, several pheophytins were produced after heat treatment, and boiling persistently reduced the antioxidant activity of peppers [39]. Pheophytin produced during 100 °C high-temperature treatment may be the reason for the decrease in antioxidant activity value of chlorophyll samples. The results showed that although heat treatment in an acidic environment would reduce the antioxidant activity value of chlorophyll samples, synergistic treatment inhibited the decrease to a certain extent.

#### 3.5.3. FTIR Analysis

As can be seen from Figure 3B, similar to the FTIR results of chlorophyll samples treated with Zn^2+^ and HHP, the FTIR patterns of chlorophyll samples remained unchanged compared with untreated samples after co-treatment and 100 °C thermal treatment. It can be concluded that thermal treatment (100 °C) in an acidic environment (pH = 5.5) does not destroy the internal structure of chlorophyll and Zn-chlorophyll. Some studies have shown slight differences in the FTIR spectra of chlorophyll and Zn-chlorophyll [32,33]. However, Mathiyalagan et al. [22] extracted and purified chlorophyll from green leaves and then synthesized Zn-chlorophyll, and the FTIR spectrum of Zn-chlorophyll was very similar to that of chlorophyll. This discrepancy in results may be due to the different substrates on which the experiments were based, our experiments were based on standard purified substances and on food.

#### 3.5.4. Fluorescence Scanning Analysis 

**Figure 5:**
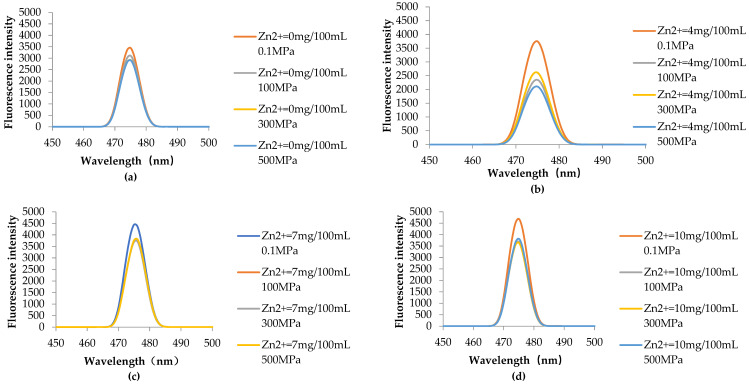
Fluorescence emission spectrum of samples after 100 °C thermal treatment at different concentrations of Zn^2+^ and high hydrostatic pressure (HHP) treatment. (**a**–**d**) are 0.1, 100, 300 and 500 MPa treated samples, respectively.

From Figure 5, when the pressure was 0.1 MPa, the fluorescence intensity of chlorophyll samples showed an increasing trend with the increase of Zn^2+^ concentration (0, 4 7, and 10 mg/100 mL). After Zn^2+^ replacement with synergistic HHP treatment and 100 °C −10 min thermal treatment, it can be intuitively observed that the application of pressure causes the fluorescence quenching of chlorophyll samples. When the Zn^2+^ concentration is 0 and 4 mg/100 mL, it can be clearly seen that the fluorescence intensity of chlorophyll samples decreased with the increase of pressure. However, when the Zn^2+^ concentration is 7 and 10 mg/100 mL, although it can be observed that the pressure causes the fluorescence quenching of the sample, there is no significant difference between 100, 300, and 500 MPa. The fluorescence quenching of chlorophyll is attributed to the decline of its intermolecular distance, while the interactions between chlorophyll molecules hinder its fluorescence release [37]. HHP acts on intermolecular interactions rather than covalent bonds between atoms in molecules [40]. Therefore, HHP has little effect on the molecular structure of chlorophyll, and does not affect the covalent bond between chlorophyll molecules [41]. Li et al. [23] also obtained the same result demonstrating chlorophyll fluorescence quenching occurred when a pressure of 600 MPa was applied to the chlorophyll solution added with 7.8% sodium chloride and after 80 °C −15 min thermal treatment.

## 4. Conclusions

The color, antioxidant activity, Zn^2+^ replacement rate, and thermal stability of the chlorophyll samples were greatly improved after the synergistic effect of Zn^2+^ and HHP. Under the synergistic effect of Zn^2+^ and HHP, the tolerance of chlorophyll to heat and acid was greatly improved. Because Zn-chlorophyll is a substance with a more stable structure, it can still maintain its bright green color in extreme environments (acid and 100 °C high temperature). HHP can promote the aggregation of chlorophyll, and aggregation can enhance the stability of chlorophyll. When the Zn^2+^ is 10 mg/100 mL and the pressure is 500 MPa, the −a* value of the sample is the largest. Furthermore, they remain bright green even after 10 min of thermal stability treatment at 100 °C. The fluorescence quenching in HHP fluorescence online analysis shows that in an acidic environment, due to the intolerance of chlorophyll to acid, magnesium ions are replaced by hydrogen ions, and chlorophyll becomes pheophytin. In the process of HHP treatment, HHP may be able to promote Zn^2+^ to enter the central structure of chlorophyll and form Zn-chlorophyll. This provides a new idea for the non-thermal promotion of chlorophyll stability. This study described the green protection mechanism of Zn^2+^ and HHP, broadening the horizon for the development of color protection in vegetable processing. It is expected to replace or supplement traditional color protection methods in frozen vegetables, beverages, canned foods and other food applications.

## Figures and Tables

**Table 1:** Replacement rate of Zn^2+^ of samples treated with different concentrations of Zn^2+^ and high hydrostatic pressure (HHP) treatment.

Sample	Chlorophyll *a* (mg/100 mL)	Chlorophyll *b* (mg/100 mL)	Pheophytin *a* (mg/100 mL)	Pheophytin *b* (mg/100 mL)	Zn^2+^ Replacement Rate (%)
Zn^2+^ = 0 mg/100 mL 0.1 MPa	2.13 ± 0.01	2.03 ± 0.03	0.56 ± 0.07	0.74 ± 0.17	0.00 ± 0.00 ^c^
Zn^2+^ = 0 mg/100 mL 100 MPa	3.75 ± 0.27	2.04 ± 0.01	0.35 ± 0.06	0.87 ± 0.09	0.00 ± 0.00 ^c^
Zn^2+^ = 0 mg/100 mL 300 MPa	2.16 ± 0.05	1.99 ± 0.02	0.45 ± 0.03	1.16 ± 0.16	0.00 ± 0.00 ^c^
Zn^2+^ = 0 mg/100 mL 500 MPa	2.15 ± 0.02	2.09 ± 0.02	0.42 ± 0.02	1.00 ± 0.19	0.00 ± 0.00 ^c^
Zn^2+^ = 4 mg/100 mL 0.1 MPa	4.20 ± 0.90	2.18 ± 0.02	0.70 ± 0.18	1.13 ± 0.25	16.03 ± 9.43 ^b^
Zn^2+^ = 4 mg/100 mL 100 MPa	3.35 ± 1.08	2.05 ± 0.04	0.43 ± 0.01	0.89 ± 0.00	27.24 ± 8.45 ^ab^
Zn^2+^ = 4 mg/100 mL 300 MPa	2.99 ± 0.85	2.09 ± 0.08	0.44 ± 0.07	0.67 ± 0.17	30.85 ± 7.08 ^ab^
Zn^2+^ = 4 mg/100 mL 500 MPa	2.13 ± 0.03	2.06 ± 0.03	0.43 ± 0.06	0.74 ± 0.24	39.31 ± 1.20 ^ab^
Zn^2+^ = 7 mg/100 mL 0.1 MPa	2.63 ± 0.50	2.04 ± 0.04	0.38 ± 0.05	0.68 ± 0.20	36.94 ± 5.32 ^ab^
Zn^2+^ = 7 mg/100 mL 100 MPa	2.15 ± 0.01	2.03 ± 0.09	0.53 ± 0.04	0.85 ± 0.21	38.15 ± 2.99 ^ab^
Zn^2+^ = 7 mg/100 mL 300 MPa	2.22 ± 0.04	2.03 ± 0.03	0.56 ± 0.09	1.15 ± 0.19	33.73 ± 0.96 ^ab^
Zn^2+^ = 7 mg/100 mL 500 MPa	2.50 ± 0.27	2.03 ± 0.00	0.34 ± 0.07	0.73 ± 0.12	37.82 ± 0.92 ^ab^
Zn^2+^ = 10 mg/100 mL 0.1 MPa	2.13 ± 0.02	2.01 ± 0.11	0.64 ± 0.34	0.83 ± 0.31	37.63 ± 8.72 ^ab^
Zn^2+^ = 10 mg/100 mL 100 MPa	2.26 ± 0.07	2.00 ± 0.01	0.44 ± 0.08	0.97 ± 0.33	36.94 ± 5.32 ^ab^
Zn^2+^ = 10 mg/100 mL 300 MPa	2.14 ± 0.04	2.06 ± 0.01	0.47 ± 0.03	0.77 ± 0.29	39.66 ± 3.07 ^ab^
Zn^2+^ = 10 mg/100 mL 500 MPa	2.16 ± 0.05	2.02 ± 0.02	0.46 ± 0.02	0.55 ± 0.03	42.34 ± 0.80 ^a^

Different letters in the same column indicate significant difference at *p* < 0.05. Values are means ± standard deviation.

## Data Availability

The data presented in this study are available on request from the corresponding author.

## Supplementary Materials

The following supporting information can be downloaded at: www.mdpi.com/article/10.3390/foods11142129/s1, Figure S1: Effects of different Zn^2+^ concentrations and high hydrostatic pressure (HHP) treatment on the appearance of chlorophyll sample solution.; Figure S2: HPLC spectra of chlorophyll samples at 665 nm after different Zn^2+^ concentrations and high hydrostatic pressure (HHP) treatment.
