# Supplementary material for: Effects of Different Zn2+ Concentrations and High Hydrostatic Pressures (HHP) on Chlorophyll Stability
Source: Foods. 2022 Jul 18;11(14):2129. doi: 10.3390/foods11142129 (PMC9316298; doi:10.3390/foods11142129)
Supplement: Supplementary file 1 [file foods-11-02129-s001.zip › foods-1773065-supplementary.pdf]

Supplementary Materials

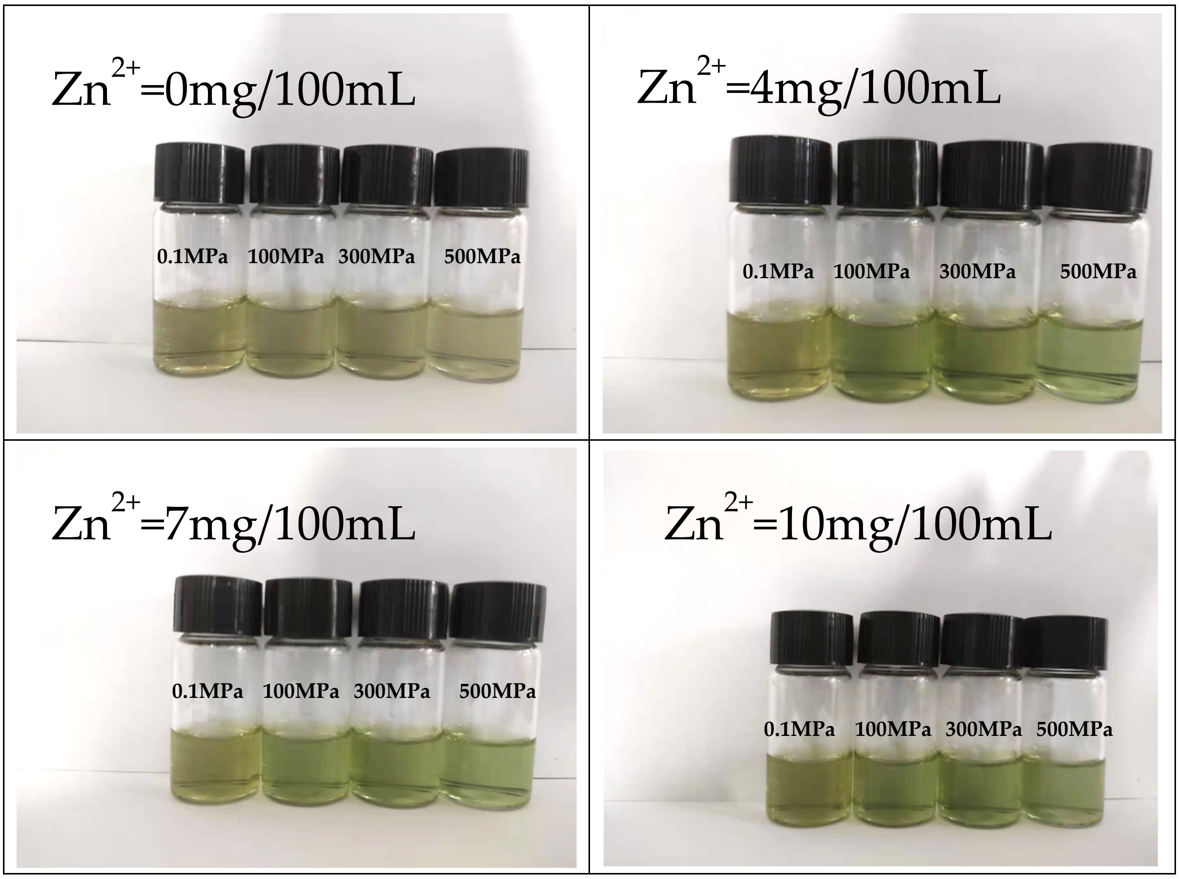

Figure S1. Effects of different Zn^{2+} concentrations and high hydrostatic pressure (HHP) treatment on the appearance of chlorophyll sample solution.

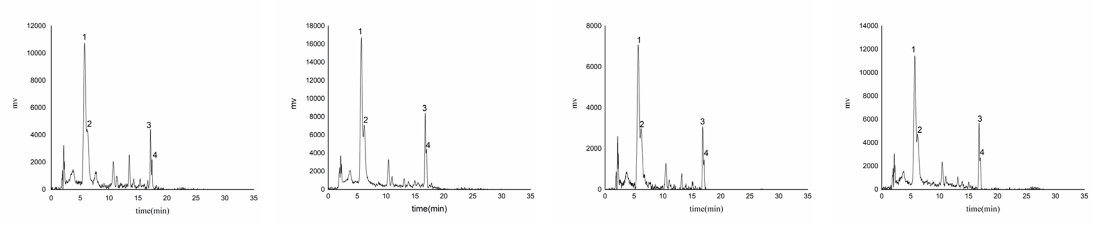

a b c d

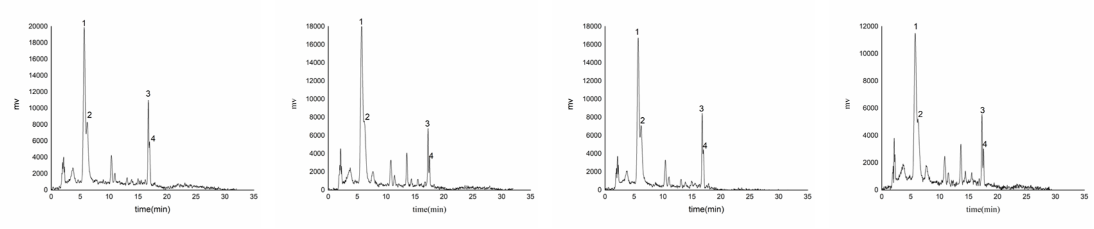

e f g h

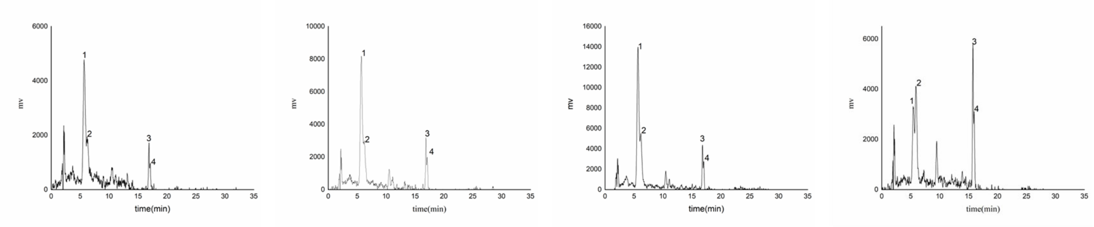

i j k l

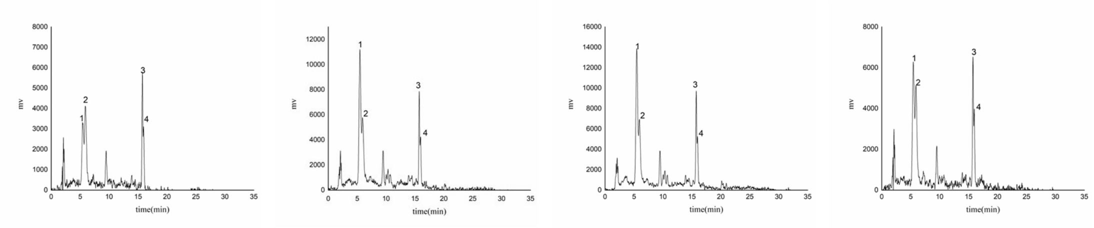

m n o p

Figure S2. HPLC spectra of chlorophyll samples at 665nm after different Zn^{2+} concentrations and high hydrostatic pressure (HHP) treatment.

* a, b, c, and d respectively represent the samples treated at 0.1, 100, 300, and 500MPa when the Zn^{2+} concentration is 0mg / 100mL, e, f, g, and h respectively represent the samples treated at 0.1, 100, 300, and 500MPa when the Zn^{2+} concentration is 4mg / 100mL, i, j, k, and l respectively represent the samples treated at 0.1, 100, 300, and 500MPa when the Zn^{2+} concentration is 7mg / 100mL, m, n, o, and p respectively represent the samples treated at 0.1, 100, 300, and 500MPa when the Zn^{2+} concentration is 10mg / 100mL. Peak 1 is the chlorophyll *b*, Peak 2 is the chlorophyll *a*, Peak 3 is the pheophytin *b*, and Peak 4 is the pheophytin *a*.
